# Pattern of Tumor-Infiltrating Lymphocytes in Mixed Epithelial and Stromal Tumor of the Kidney: A Review of Five Cases

**DOI:** 10.3390/cells10040917

**Published:** 2021-04-16

**Authors:** Hye Won Lee, Hyunwoo Lee, Chanho Park, Won Joon Oh, Tae Jin Kim, Ghee Young Kwon, Seong Il Seo

**Affiliations:** 1 Department of Urology, Center for Urological Cancer, National Cancer Center, Goyang 10408, Korea; uroproper@ncc.re.kr; 2 Department of Pathology and Translational Genomics, Samsung Medical Center, Sungkyunkwan University School of Medicine, Seoul 06351, Korea; hwpatho.lee@samsung.com; 3 Department of Immunology, Sungkyunkwan University School of Medicine, Suwon 16419, Korea; darkstar0113@hanmail.net (C.P.); joon516@skku.edu (W.J.O.); tjkim@skku.edu (T.J.K.); 4 Department of Urology, Samsung Medical Center, Sungkyunkwan University School of Medicine, Seoul 06351, Korea

**Keywords:** flow cytometry, kidney neoplasms, lymphocytes, tumor-infiltrating, tumor microenvironments

## Abstract

Mixed epithelial and stromal tumor of the kidney (MESTK), a benign rare tumor with malignant transformation potential, is thought to be derived from fetal or immature cells originating from the mesonephric and Müllerian ducts. However, due to its rarity, little is known about the anti-tumor immune responses in MESTK. Herein, we present five cases of MESTK and evaluate the population of tumor-infiltrating lymphocytes (TILs) using a freshly obtained MESTK sample. Microscopically, TILs were scattered or clustered in large aggregates in the stroma in all five cases; furthermore, three cases exhibited heavy, large lymphocytic aggregates with no well-organized tertiary lymphoid structures with germinal centers. Flow cytometric analysis of TILs in one freshly obtained MESTK sample revealed that >40% of CD3^+^ T cells were effector memory Fas^+^CD28^−^ γδ T cells expressing high levels of programmed cell death protein 1 and inducible T-cell co-stimulator, but low levels of CD44 and CD27. Most αß T cells exhibited a naïve phenotype. Additionally, we detected many activated class-switched CD21^+^CD27^+^ B cells as well as CD11c^high^IgM^high^ marginal zone B-like and CD27^−^CD21^−^CD23^−^ immunoglobulin (Ig)D^high^IgM^low^ age-associated B-like cells. Collectively, for the first time, we report the immune microenvironment pattern of MESTK to oncogenic stress.

## 1. Introduction

Mixed epithelial and stromal tumor of the kidney (MESTK) is a unique renal neoplasm characterized by epithelial and stromal proliferation with variable cellularity and growth patterns [1,2,3,4]. MESTK has been described as a Bosniak category III or IV mass mimicking renal-cell carcinoma and multicystic dysplastic kidney [2,3]. The stromal component exhibits areas that range from paucicellular and fibrous, ovarian-like, solid, and leiomyomatous to myxoid and degenerative, whereas the epithelial component exhibits a tubular, microcystic, macrocystic, or complex branching architecture [1,2,3,4]. Several reports have described that carcinomatous or sarcomatous transformation of MESTK leads to a dismal prognosis [5,6,7], suggesting that MESTK cases in which neoplastic components possess malignant potential undergo dynamic evolution. Notably, the stromal and epithelial components of MESTK are thought to be derived from a common clone with multiple differentiation potentials [8]. Particularly, higher prevalence rates of MESTK in women and a history of long-term sex steroid exposure in both sexes, combined with the presence of an ovarian-type mesenchymal component and the expression of estrogen and progesterone receptors in the ovarian-type stroma, indicate that the stromal and epithelial components originate from Müllerian remnants (paramesonephric ducts) misplaced during embryogenesis and are significantly influenced by steroid hormones during the development and progression of MESTK [1,2,3,4,9].

Intratumoral immune infiltrates comprise tumor-associated macrophages, dendritic cells, mast cells, granulocytes, and natural killer (NK) cells from the innate immune system and B cells, CD4^+^ and CD8^+^ T cells, and NK T cells from the adaptive immune system as well as γδ T cells, which belong somewhere between the innate and adaptive immune systems [10]. The composition of these infiltrates is determined by tumor cell- and tumor microenvironment-derived signals, as well as gene expression that is specific to the normal tissue of origin. Tumor-infiltrating lymphocytes (TILs) have been the focus of attention for decades in anticancer research due to their clinicopathologic significance, i.e., their utility in adoptive immunotherapy. Recently, with the advent of immunotherapy, the significance of TILs has been re-appraised as a component of the tumor microenvironment which affects the efficacy of immune checkpoint blockade [10]. As little is known about the antitumor immune response against MESTK compared with other malignancies of the kidney, we reviewed the immunopathologic characteristics of MESTK in five patients with this tumor and performed an in-depth analysis of the phenotype of TILs using a freshly obtained MESTK specimen from one of the patients.

## 2. Materials and Methods

### 2.1. Patients and Samples

Five cases diagnosed with MESTK during the period from 2015 to 2018 were retrieved from the archives of the Department of Pathology at Samsung Medical Center (Seoul, Korea). The present study protocol was reviewed and approved by the Institutional Review Board of Samsung Medical Center (approval number: 2018-04-037). Informed consent was obtained by all subjects when they were enrolled.

The diagnosis of MEST was established according to standard criteria [1,11]. All of the resected tumor specimens were formalin fixed, paraffin embedded, sectioned and stained with hematoxylin and eosin. The hematoxylin-eosin stained slides were reviewed by three pathologists (G.-Y.K., T.-J.K. and H.-W.L. (Hyun-Woo Lee)) to confirm the diagnosis. We reviewed gross features and histologically assessed the presence or absence of smooth muscle differentiation, hypocellular collagenized fibrous stroma, hypocellular edematous stroma, cellular stroma, and wavy spindle cell stroma in each tumor. Detailed histologic features of the MESTK samples including the thickness of the cystic septa, percentage of stromal cells, stromal cellularity, presence of ovarian-type stroma, smooth muscle metaplasia, and complex branching glands were evaluated. In addition to the flat, cuboidal, and hobnail epithelium lining the cysts, we examined the presence of unusual epithelial components, including ciliated cells, urothelium, clear cells, squamous differentiation, and mucinous goblet cells. Other features recorded included calcification, ossification, hemosiderin-bearing histiocytes, and inflammation.

### 2.2. Immunohistochemistry

Immunohistochemical (IHC) staining was performed on representative 4-μm-thick formalin-fixed, paraffin-embedded (FFPE) tumor tissue blocks for the five cases using the Leica BOND-MAX™ system (Leica Biosystems, Wetzlar, Germany) according to the manufacturer’s protocol. We used antibodies for the ER (6F11, 1:300, Novocastra, New Castle, United Kingdom, 1:300), PR (clone 16, Novocastra, 1:1200), premelanosome protein (HMB-45, Dako, Carpinteria, CA, USA, 1:80), cytokeratin (PAN CK) (AE1/AE3, Dako, 1:500), CD 20 (L26, Novocastra, 1:800), CD 3 (polyclonal, Dako, 1:300), leukocyte common antigen (CD 45 RB) (2B11 + PD7/26, Dako, 1:1000) and CD 56 (CD564, Novocastra, 1:200).

### 2.3. Flow Cytometric Analysis

To analyze lymphoid populations within the tumor from one patient (patient 4), the fresh tumor tissue was finely minced with scissors and filtered through a nylon membrane, and a single-cell suspension was purified over a Ficoll–Hypaque density gradient. Cells were stained with the following anti-human antibodies: anti-αβTCR (IP26, eBioscience, San Diego, CA, USA), anti-CD8 (OKT8, eBioscience), anti-CD44 (IM7, eBioscience), anti-PD-1 (eBioJ105, eBioscience), anti-CD27 (O323, eBioscience), anti-CD20 (2H7, eBioscience), anti-CD1c (L161, eBioscience), anti-γδTCR (B1, BioLegend, San Diego, CA, USA), anti-CD4 (OKT4, BioLegend), anti-CD28 (CD28.2, BioLegend), anti-FAS (DX2, BioLegend), anti-ICOS (C398.4A, BioLegend), anti-IgM (MHM-88, BioLegend), anti-CD21 (B-ly4, BD Biosciences, San Jose, CA, USA), anti-CD23 (M-L233, BD Biosciences), and anti-IgD (Southern Biotech, Birmingham, AL, USA). Flow cytometry was performed using a BD FACS Canto II flow cytometer (BD Biosciences) and analyzed via FlowJo software (Tree Star, Ashland, OR, USA).

## 3. Results

### 3.1. Clinicopathologic Characteristics and Immunoreactivity of the Five Cases of MESTK

The clinicopathologic features of the five patients with MESTK are summarized in Table 1. All five patients underwent partial nephrectomy.

**Figure 1:**
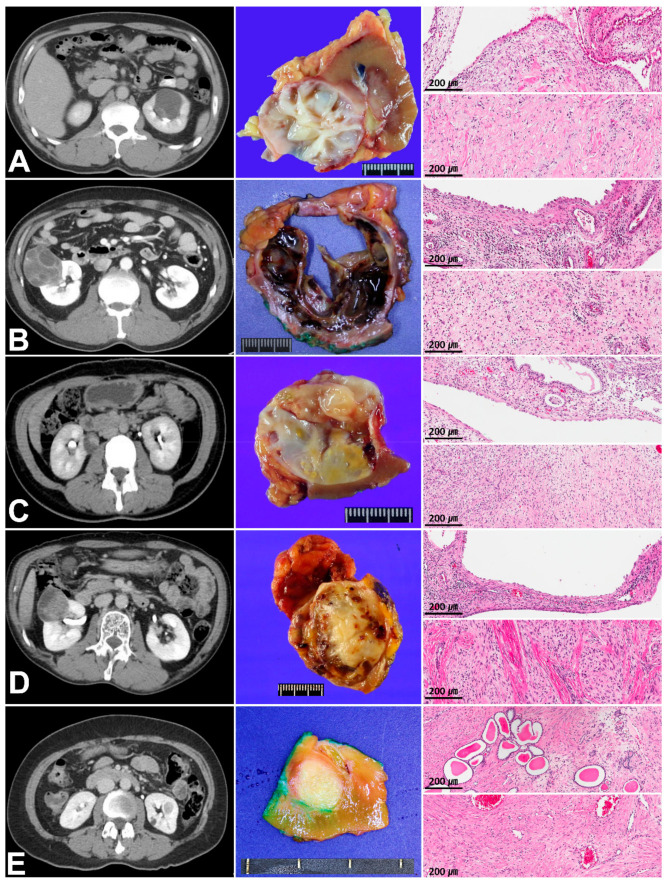
Images of abdominal computed tomography, gross pathology, and histology of the five patients with mixed epithelial and stromal tumor of the kidney (MESTK). Representative images from the five cases reviewed in this study, including CT (left panel), photograph of a cut section of the gross specimen (middle panel), and histopathologic images of the epithelial and stromal regions of MESTK. (**A**), case 1; (**B**), case 2; (**C**), case 3; (**D**), case 4; and (**E**), case 5.

**Figure 2:**
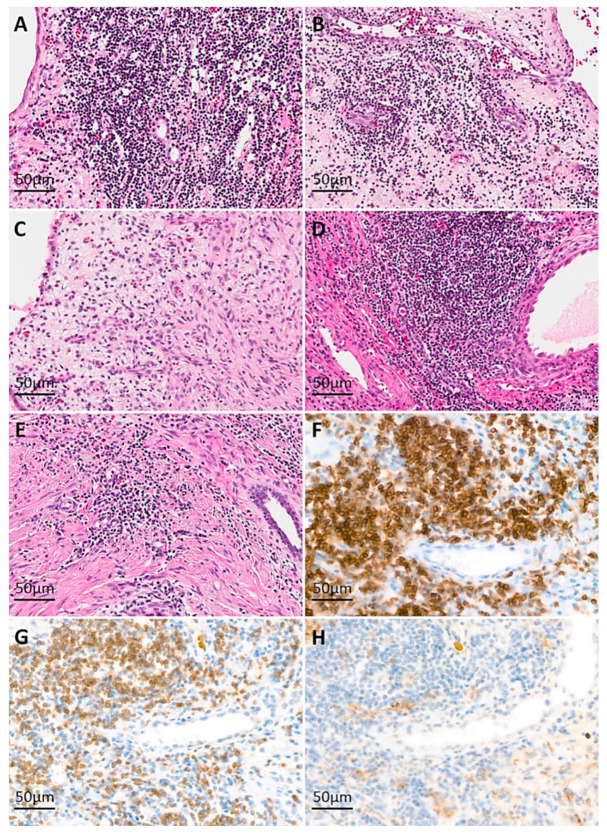
Immune cell infiltration into mixed epithelial and stromal tumor of the kidney (MESTK). ((**A**), case 1, hematoxylin and eosin (H&E), original magnification ×100; (**B**), case 2, H&E, ×100; (**C**), case 3, H&E, ×100; (**D**), case 4, H&E, ×100; (**E**), case 5, H&E, ×100) Lymphocytic infiltration is seen around blood vessels within the stromal areas in cases 1, 2, 4, and 5 ((**B**–**F**), respectively). In case 3, loose stromal area shows scattered lymphocytic infiltration (**D**). Immunohistochemical staining for evaluation of the composition of lymphoid cells in case 4 ((**F**), CD 45 RB, ×100; (**G**), CD 3, ×100; (**H**), CD 56, ×100).

Grossly, all cases were well-circumscribed in common, with a mixture of solid and cystic portions (Figure 1). Microscopically, the cystic structures were lined with a single layer of flat-to-cuboidal epithelial cells. Some cases showed epithelial cells with hobnail features, but obviously malignant epithelial cells were not identified in any cases. The solid component mainly contained various amounts of proliferating fibroblasts. The variable patterns of lymphocytic infiltration were optimized in Figure 2. Case 1 and 4 showed heavy lymphocytic infiltrating foci, especially in the periphery of the tumor (Figure 2A,B, arrows) and case 2 showed multifocal collections of lymphocytes with no GCs, accompanied with a spindle cell component (Figure 2C). The lymphocytic pattern of case 3 was similar to case 2, but much fewer lymphocytes were present in case 3. Unlike case 2 and 3, which showed patterns of lymphocytes located around the spindle cell component, case 5 revealed that lymphocytes were present focally adjacent to the epithelial structures. In immunohistochemical staining, the lymphocytes were composed of similar proportions of T and B cells. The population of NK cells was not prominent (Figure 2H). The epithelial cells were commonly positive for pan-cytokeratin, and variable portions of spindle cells were positive for PR.

### 3.2. Flow Cytometric Analysis of Intratumor Lymphocytes

**Figure 3:**
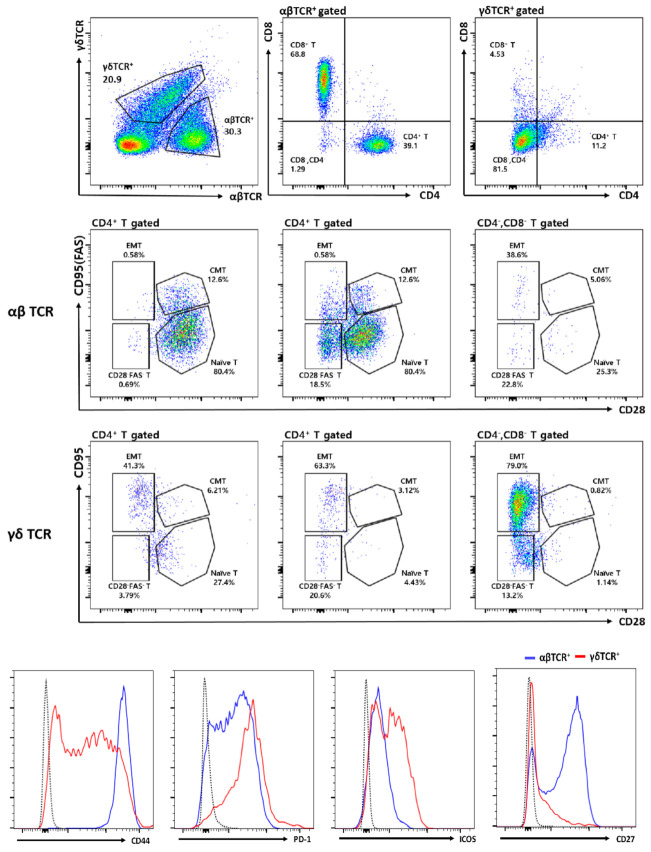
Analysis of tumor-infiltrating T cells in case 4. (A) Flow cytometry plots displaying effector memory T cells (EMT, CD28^−^FAS^high^), central memory T cells (CMT, CD28^+^FAS^+^), naïve T cells (CD28^+^FAS^−^), and CD28^−^FAS^−^ T cells among αβ T or γδ T cells in the mixed epithelial and stromal tumor of the kidney (MESTK) tissue. The histogram at the bottom compares the expressions of CD44, programmed cell death protein 1 (PD-1), inducible T cell costimulatory-1 (ICOS-1), and CD27 between αβ T (blue) and γδ T cells (red). Broken gray lines denote FMO control.

In all five cases, lymphocytes were scattered or clustered as large aggregates in the stroma. However, neither intratumoral tertiary lymphoid structures (TLS) nor well-formed GCs were observed in any case. We surmised that these results may indicate the presence of innate-like immune responses, specifically against stromal tumor cells [12]. To investigate the specific lymphocyte populations in MESTK, we analyzed the composition of TILs in a freshly isolated tumor sample from patient 4 using fluorescence-activated cell sorting. Interestingly, ~40% of the CD3^+^ T cells were γδ T cells (ratio of γδ to αβ T cells being approximately 2:3) showing an effector memory Fas^+^CD28^−^ phenotype that is equivalent to a previously defined CD27^−^CD45RA^−^ γδ T-cell subset [13] (Figure 3). Most γδ T cells were negative for both CD4 and CD8 and expressed high levels of programmed cell death protein 1 and inducible T-cell costimulator, but low levels of CD44 and CD27. In contrast, most αβ T cells were naïve CD4^+^ or CD8^+^ T cells.

**Figure 4:**
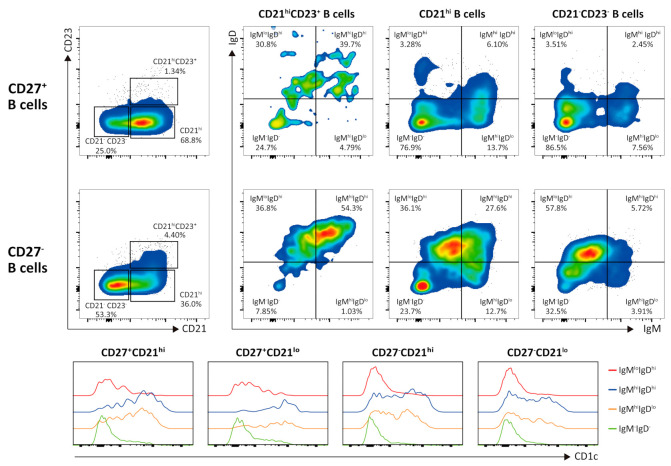
Analysis of tumor-infiltrating B cells in case 4. Flow cytometry plots show B cell subsets in the MESTK tissue. B cells subsets were gated according to CD21, CD23, IgM, IgD, and CD27 expression levels. The expression of CD1c in each B cell subset is shown in the histogram.

B cell infiltration was also prominent, but the composition of B cells in the tumors was quite distinctive (Figure 4). In accordance with the lack of organized tertiary lymphoid tissues, most B cells were not follicular, as they exhibited low or no expression of CD23. Similar numbers of B cells were found to be negative and positive for the expression of CD27, a known marker of memory B cells. Most of the CD27^+^ B cells were class-switched IgM^−^IgD^−^ B cells (as shown in flow cytometry plots for CD21^high^B cells and CD21^−^CD23^−^B cells), but CD27^+^IgM^high^ B cells also expressed a high level of CD1c (as shown in histogram), resembling the phenotype of marginal-zone B cells (MZBs). In contrast, most of the CD27^−^CD21^high^ B cells expressed a high level of IgD, suggesting that they were naïve follicular B cells; however, their low CD23 expression was distinct from that of most circulating follicular B cells. Notably, half of the CD27^−^ B cells expressed low CD21 and CD23 levels and were IgD^high^ and class-switched IgM^−^IgD^−^, resembling the phenotype of age-associated B cells (ABCs) [14].

## 4. Discussion

Under homeostatic conditions, the kidney contains only a small number of immune cells including dendritic cells and macrophages as well as a few lymphocytes [15]. In contrast, cells from the adaptive and innate immunity systems infiltrate tumors, as shown in a study investigating the immune landscape in clear cell renal cell carcinoma [16]. This study demonstrated an association among the tumor-associated macrophage phenotype, regulatory T cells, and immunosuppressed CD8^+^ T cells. TILs often form simple lymphocytic aggregates or more complex structures that resemble TLS [12], with a spatial organization comprising compartmentalized B- and T-cell-rich zones that allows direct interaction between B and T cells and GC reaction within the tumor [12]. In addition, cancer-associated fibroblasts (CAFs; myofibroblasts), originating from the activated resident fibroblasts in the renal interstitium [17], serve to recruit and educate a variety of immune cells as the predominant subpopulation within the tumor microenvironment. This finding suggests the importance of the interaction between CAFs and tumor-infiltrating immune cells for establishing specific immune system-related anticancer effects or a tumor-permissive environment [18]. Thus, cancer entities such as MESTK that comprise a CAF-rich stromal component may be more susceptible to antitumor immunity mediated by activated TILs that are recruited by CAFs.

The epithelial component of MESTK is interspersed throughout the mesenchymal components, and both stromal and epithelial cells may be neoplastic and derived from the same progenitor cells [8,9]. As mesonephric and Müllerian ducts share a common basement membrane in urogenital ridges in the fourth week of development, ovarian stromal cells can become incorporated into the ureteric bud and metanephric mesoderm [19]. In MESTK, hormone stimulation or imbalance may activate the misplaced immature mesenchyme that harbors the capacity for dual epithelial and mesenchymal differentiation, leading to the secretion of paracrine factors that induce changes in the adjacent epithelium. Moreover, the neoplastic stroma in MESTK may entrap tubules, stimulating their growth and leading to cyst formation, which further highlights the association between renal obstructive changes and reactive Müllerian metaplasia in the renal ovarian-like stroma in MESTK, suggesting a stromal–epithelial interaction [20]. Therefore, tumor-associated ligands sensed by γδ T cells in MESTK may be unidentified oncofetal antigens (OFAs) expressed by tumors, such as teratoma, which contain structures that resemble the ureteric bud and metanephric mesoderm tissues in MESTK. Pronounced immune responses were observed within the tumor and peripheral blood of patients with teratoma, as indicated by the appearance of the anti-N-methyl-D-aspartate receptor antibody [21]. In particular, OFAs expressed in normal developing fetal tissues but not in adult tissues are actively re-expressed universally at high levels in tumor and cancer cells spontaneously arising from other oncogenic mutations, as well as in cancers induced by viruses, carcinogens, and radiation [21]. Although the presence of oncofetal antigens (OFAs) associated with Müllerian remnants might lead to sustained activation of the innate and adaptive immune systems in the MESTK microenvironment, to date, the immune responses against MESTK cells are not well understood.

Here, we observed prominent lymphocytic cell aggregation distinct from TLS in five MESTK cases. Furthermore, flow cytometry of TILs in case 4 showed an interesting pattern of immune cell activation characterized by the abundance of innate-like γδ T cells with an effector memory phenotype as well as activated B cells resembling MZBs, ABCs, and class-switched B cells. Interestingly, most CD4^+^ and CD8^+^ αβ T cells were of naïve phenotype in contrast to γδ T or B cells. In the peripheral blood, αβ T cells account for approximately 95% of the CD3^+^ cells, whereas γδ T cells account for only 5%.

Increasing evidence indicates that human γδ T cells play an important role in the early first-line defense against exogenous pathogens, endogenous damage, and oncogenic stress-induced ligands [22,23]. This response occurs without prior priming, in a major histocompatibility complex-unrestricted manner, linking innate and acquired immunities [22,23]. Stress-induced molecules are detected using T-cell, Toll-like, and NK receptors, which act separately or synergistically to activate specific γδ T cell effector functions [22,23]. Activated γδ T cells secrete chemokines, such as chemokine ligand 3, chemokine ligand 4, chemokine (C-X-C motif) ligand (CXCL) 10, and CXCL13, to recruit αβ T cells, B cells, NK cells, and macrophages/dendritic cells to the tumor site [23,24]. Activated γδ T cells exhibit profound antitumor immunity, for which they utilize death receptor/ligand-dependent and cytotoxic effector molecules, such as perforin and granzyme, in addition to mounting a T helper type 1 response [23,24]. As professional antigen-presenting cells, γδ T cells also promote conventional T cell responses to antigens and assist B cells in producing IgA, IgM, and IgG antibodies [23,24]. Intratumoral γδ T cells are likely the most important favorable prognostic immune population because of their response to stress signals from actively proliferating tumor cells in a variety of tumors, including RCC [23,24,25]. Based on the results of the present study, we suggest that heavy infiltration of effector memory γδ T cells can contribute to a favorable MESTK prognosis because of the profound anticancer immune responses of these cells to stress signals expressed by tumor cells. γδ T cells recognize cancer cells by detecting isopentenyl pyrophosphate, which is generated via the mevalonate pathway and accumulates during dysregulated metabolism in cancer cells [23,24,25]. Furthermore, potent γδ T cell activation can be mediated by damage-associated molecular patterns (DAMPs) such as mitochondrial DNA and high mobility group box 1 protein via pattern recognition receptors such as Toll-like receptors [23,24,25]. Correspondingly, dysregulated proliferation and malignant transformation of the epithelial and stromal components of MESTK could be sensed as DAMPs by γδ T cells, consistent with previous reports stating that oncogenic stress in renal tubular cells is sensed by the immune system via cell-cycle arrest and apoptosis induction [23,24,25,26].

In contrast, tumor-infiltrating B lymphocytes may exert multiple antitumor effects, through antigen presentation and cytokine production, that enhance the cytotoxic activity of T cells [27]. Furthermore, B lymphocytes may exert tumoricidal effects directly through the secretion of granzyme B or indirectly through antibody-dependent mechanisms [27]. Notably, large numbers of class-switched, MZB- and ABC-like cells in the ovarian-like stroma of MESTK have antitumor activity, as observed in previous studies reporting that the presence of memory B cells primarily in infiltrated lymphoid structures in the stroma of high-grade serous ovarian cancer correlates with a better prognosis [27,28]. Our flow cytometric analysis showed that approximately 70% of CD27^+^ B cells expressed CD21. Although most CD27^+^CD21^+^ B cells were class-switched IgM^−^IgD^−^ B cells, approximately 20% of CD27^+^CD21^+^ B cells were non-switched B cells. Interestingly, we observed that the non-switched CD27^+^CD21^+^ B cells expressed CD1c, which is a feature of human circulating MZBs [29]. It remains to be determined whether these MZB-like cells reflect the infiltration of blood MZBs or the tumor microenvironment-induced in situ expression of CD1c. Follicular B cells within the tumors did not express CD23. We speculate that the low CD23 expression may reflect the poor formation of TLS, as CD23 expression is induced in the lymph node follicular environment.

Notably, we also observed ABC-like cells within MESTK. ABCs are a newly discovered, phenotypically and functionally unique subset of CD11c^+^ B cells that express low levels of CD21 and CD23 and elevated levels of CD5, Fas, and CD138; furthermore, they express several markers shared with exhausted memory B cells and the transcription factor T-bet [14,30]. Although ABCs display a characteristic transcriptional profile, homeostatically competing with naïve follicular cells and MZBs, they respond to Toll-like receptor 9 and, to a lesser degree, Toll-like receptor 7 ligation instead of responding to B-cell receptor and CD40 ligation, indicating that they are refractory to activation via adaptive immune receptors yet responsive to innate receptor stimulation [30]. The accumulation of ABCs in the absence of TLS appears to reflect a highly inflammatory tumor microenvironment.

In summary, we reported a unique immune response against MESTK that is characterized by the infiltration of γδ T cells and activated B cells without TLS and GC reaction. Our study was limited by the lack of fresh MESTK samples owing to the rarity of this neoplasm. However, our observation of TILs in one case of MESTK led us to review other MESTK cases, and thus, to the confirmation of similar immune response in most MESTK cases. In future studies, it would be interesting to investigate the unique OFAs recognized by γδ T cells and the possible relationship between activated B cells such as ABCs and MZBs and activated γδ T cells.

## Figures and Tables

**Table 1:** Clinicopathologic characteristics of patients.

Variables	Number
Sex	
Male	3
Female	2
Age (years)	51.4 (34–60)
History of Hormone therapy	
Absent	4
Present	1
Tumor size (mm)	37.6 (18–55)
Recurrence	
Absent	5
Survival status	
Alive	5
Follow-up period (months)	9.3 (1.1–27.2)

Data are presented as mean (range) unless otherwise noted.

## Data Availability

The data and materials are available from the author.
